# “I Dressed Him, God Cured Him”: Ambroise Paré, the Father of Surgery

**DOI:** 10.7759/cureus.72717

**Published:** 2024-10-30

**Authors:** Krishna B Bhadriraju, Robert Goodwin

**Affiliations:** 1 Research, Jenks High School, Jenks, USA; 2 Trauma Institute, Saint Francis Health System, Tulsa, USA

**Keywords:** biography, historical vignette, history, surgery, traumatology

## Abstract

Considered the father of surgery, Ambroise Paré was a French military surgeon who creatively improvised and used various surgical instruments during his era. He believed in the principle of evidence-based medicine to treat his patients. He advocated for ligature instead of cauterization in surgeries. He left an incredible mark in the fields of modern anatomy, surgery, neurosciences, and neurotrauma. His legacy is unparalleled and his innovations unmatched in the field of surgery to date. He died peacefully at the age of 80 years at his residence in Paris. The continued reference to date of Paré’s techniques, which are now more improvised with time, is a testament to his “thinking way ahead of his times” innovations. His patient-centric approach to relieving the pain and suffering of the injured remains the legacy of this superhuman.

## Introduction and background

**Figure 1:**
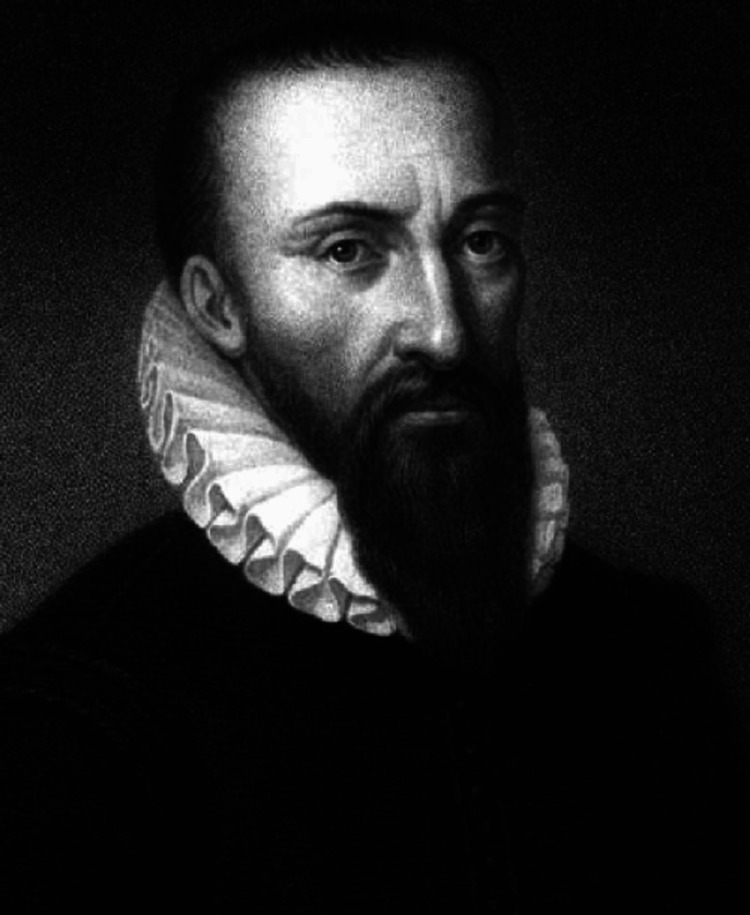
A portrait of Ambroise Paré (1510-1590) by William Holl. Source: Wikimedia Commons (Public domain) - https://commons.wikimedia.org/wiki/File:Ambroise_Par%C3%A9.jpg#filelinks.

Ambroise Paré (c. 1510-1590; Figure 1), a French military surgeon, is widely regarded as the founder of modern surgery. Bourg-Hersent, a small village near Laval in Northwest France, was the birthplace of Ambroise Paré [1-4]. His father and brother worked as barber-surgeons, a term widely used in the Middle Ages for European medical practitioners who not only attended the soldiers injured in wars but also cut hair and pulled teeth to maintain a livelihood. At one time point, Paré worked as an apprentice with his older brother and subsequently got promoted to working as an apprentice under Mr Vialat, the countess’ official barber. However, the real turning point came during Paré’s time at Hotel-Dieu Hospital, which not only helped him improve his dissecting skills but also afforded him a deeper understanding of human anatomy. Lack of funds forced him to not pursue a doctorate degree. Not to be deterred and to put his surgical skills to practical use, Paré joined the army of the Duke of Montjean. Paré practiced as a battle-field surgeon in multiple wars, which gave him first-hand experience in wound care and treatment. The creative treatment options that Paré utilized did not come from established textbooks of that era but rather from seeing the pain and suffering of soldiers in war zones. It must be recollected that established textbooks of that era were mostly Greek mythology sources. This implication is profound because Paré, as a young boy, did not read Galen’s work and other texts available during that time as he did not know Greek or Latin. Instead, it is the practical and profound experiences on the battlefield that helped him advance his knowledge of surgery and innovate in the fields of military, wound, and neurosurgeries, as well as mechanical hemostasis, pediatrics, and obstetrics. Paré’s entire legacy is archived in 25 books on anatomy and surgery, which are rich in knowledge, with concise explanations and illustrations.

## Review

While surgical treatments can be traced back to Edwin Smith Papyrus in the 17th century, these were more in the form of an encyclopedia where the accepted treatment methods during those times were mentioned. On the other hand, a wealth of knowledge came from Greece regarding healing arts and treatments, and these were a direct result of observations and analyses of patients who were treated with the same. Paré was knowledgeable of both to a certain extent, and this gave him an edge in holistically understanding a patient’s condition and the array of treatment options available.

Earliest known instance of evidence-based medicine: “I ran out of oil…..”

In the early 16th century, the conventional treatment for gunshot wounds was to boil an oil solution and pour it onto the wounds to seal them. The reasoning behind this treatment was that the solution would prevent gunpowder poisoning, a theory during those times that was not supported by evidence. This conventional method of using boiling oil as treatment was mentioned in “Wounds in General” and other books by Giovanni da Vigo [5]. Jean de Vigo’s gunshot wound management consisted of two steps: (1) cauterization with boiling oil and (2) treating the burn by using one of the many remedies such as barley decoction with earthworms, etc. [6]. In one instance when Paré was working as a military surgeon, he ran out of oil while attending to the massive number of wounded soldiers. As a result, Paré was forced to look at alternative options, which, in his opinion, had no chance of success. Fortunately, for the medical world, this creative brainstorming brought to the forefront the innovativeness of Paré. It must be recollected that Paré, as a young 24-year-old, became a military surgeon and had never seen a gunshot wound before. This essentially put his only reference source as the surgical texts available at that time. His quick thinking made him recollect the old Roman times when an ointment was applied to treat and heal gunshot wounds. Paré immediately improvised the technique and made an indigenous solution consisting of turpentine, oil of roses, and egg yolk. He applied this modified medication to the gunshot wounds of the soldiers. Paré could not sleep that night worrying if the soldiers would get treated with his new medicated solution or not [5,6].

“They had no fever…”

The next day, to his utter surprise, Paré observed that the wounds of the patients treated with the new solution were not swollen or inflamed and that the patients had a restful night. On the other hand, the other patients who were treated with boiling-oil cauterization treatment were running a fever, with complaints of pain and visible swelling in their wounds. Paré also noticed that soldiers with wounds dressed with his new treatment recuperated better than soldiers treated with boiling-oil cauterization [7,8]. This also led him to explore further the existing belief that gunpowder was toxic [1,9]. He opined that gunpowder consists of a mixture of charcoal, sulfur, and saltpetre, and, more importantly, none of these elements are poisonous by themselves. This led him to deduce that since they are not poisonous separately, they may not be poisonous as a mixture. In addition, he also recollected that German prisoners, to make their wine stronger, were putting a pinch of gunshot powder into their wine. He opined that if this addition of gunshot powder were to make the wine poisonous, the German prisoners would not continue the tradition and in fact would abandon it. Since this did not happen, Paré had further evidence that this was just a myth and was not backed by any scientific evidence. Thus, his final scientific deduction was that (a) there was no toxicity in gunpowder and (b) cauterization is not always the required treatment for gunshot wounds [1-4,10,11].

The inadvertent case-control study

Unknowingly, Paré did a two-arm case-control study, as we call it today, with some wounded soldiers receiving the “standard treatment” of boiling oil while the other wounded soldiers received the new solution. In today’s clinical research world, we would have categorized the treatment group that had Paré’s new treatment as “cases” while the old treatment group that had the traditional treatment of boiling oil cauterization would be called “controls.” After comparing the “cases” with the “controls,” Paré deduced that his new improvised medicated solution was the better treatment for gunshot wounds going forward. It must be brought to the reader’s attention that Paré did not use the term case-control study or cases vs. controls in that era.

Differential treatment

At this juncture, it is important to point out that Paré logistically and with scientific reasoning determined the differential treatment of using hot oil cauterization as a treatment plan. Some of the gunshot wounds, by their very nature, were very deep and complicated and thus resulted in gangrene. Once gangrene set in, because of the lack of present-day options, amputation was decided to be the most effective treatment. In fact, Paré was one of the first to choose a site well above the gangrenous area as the amputation site [5].

“Silk threads”

As expected, such deep wounds resulted in severe complications such as hemorrhage, infections, and sometimes even death. While not using hot oil cauterization for gunshot wounds, because of his scientific reasoning, Paré kept using it in limb amputations for hemostasis from the bone. Paré noticed that hemorrhaging was a significant challenge that needed to be addressed because of amputation. During those times, blood loss was stopped by cauterizing by iron method. In short, the heat from the red-hot cautery sealed off the blood vessels when applied. The agony caused by the heat from red-hot cautery was excruciating and this led Paré to think in terms of arterial ligation to prevent hemorrhaging with silk ligatures. Silk threads or “ligatures” were indeed a vast improvement to the vicious cauterization, but the time factor was definitely not in favor of Paré’s new improvised technique. On the battlefield, where time is of the essence, cauterizing was a fast and effective method when compared to the slow, new silk ligature technique. Moreover, Paré’s way was highly susceptible to infection while cauterization had less chance of the same, though knowledge of antibiotics was non-existent at that time. Surgeons remained skeptical of Paré’s “silk thread” technique but this provided the basis for the future work of Joseph Lister who improvised Paré’s work on ligatures by using sterilization. In his book “Dix Livres de La Chirurgie,” which he published in 1564, Paré first published his method of ligating the vessels in amputations, clearly stating that in doing so, he moved away from the cauterization method to stop bleeding [5]. This shows the righteous and just nature of Paré because he did not hesitate to inform the public that what he originally thought was the best option available did not hold true anymore, as Paré himself had recommended cauterization in his own book, published in 1552 [5,10,11].

“I dressed him, God healed him”

In 1552, while tending to the wounded in Metz, Paré amputated an officer’s leg that was broken by a cannon shot. He used his new method of ligature instead of the hot irons to stop hemorrhage. “I dressed him, God healed him” was Paré’s famous statement after the ligature method of treatment. The officer returned home in happy spirits on a wooden leg, without getting callously burnt, to stop the bleeding [5,12].

Recognizing “pain”

It must be remembered that cauterization is an extremely painful process. Paré is one of the first to recognize “pain during treatment.” In one of his works, he argued that Christians should first use less painful remedies before resorting to burning a wound by fire. An interesting observation with the above statement alludes to the fact that Paré who was a Protestant, a Huguenot in particular, maintained the appearance of being Catholic and, in fact, baptized his children in the Catholic faith. And, to state facts, Paré was buried in the Catholic faith [13].

Paré revisited “pain” when talking about surgeons. He opined that a surgeon should operate tenderly and smoothly so that damage to tissue is limited with reduced pain. This reflects the holistic as well as humanistic approach that Paré had with his patients as operating gently and less aggressively not only benefits the patient but also increases the chances of patient outcomes [1].

Innovations in neurotraumatology

In 1552, during a war in the City of Metz in France, Paré treated an unconscious and convulsing soldier who suffered a blow to the head from a stone cannonball. Paré performed skull trepanation on the soldier and applied alum and honey mixture to protect his wounds [14]. Paré demonstrated the repetitive success of this technique by performing the same procedure on other soldiers with head wounds [10]. It is indeed not an extrapolation to say that many instruments that are used in neurosurgery today are largely modernized versions of instruments used by Paré [15].

Invention of saws for use in skull surgery

Paré devised a saw that was round-shaped, and this was used for cutting out a piece of skull in the form of a circle, almost like the modern-day hand drill used in the 16th century. He designed this trepan with a lid that offers protection by minimizing harm if used by unskilled hands, thus demonstrating his in-depth thinking powers and the patient-centric thinking he had during those times. He went further ahead and to increase the stability of the drill when working on the skull, he moved from a two-point to a three-point bearing trepan [16].

Bec de corbin

Paré designed “Bec de Corbin,” which is a predecessor to modern hemostats [12].

Obstetrics

In the 16th century, Paré revived the practice of the podalic version in pregnant women. He convinced midwives and gynecologists that even in cases of head presentation, by utilizing the podalic version, surgeons could often deliver the infant safely. This is a huge milestone in maternal and child safety as the common notion in those days was when an infant showed head presentation, it may result in surgeons dismembering the baby and extracting the baby piece by piece [12,17,18]. One can only wonder how many babies Paré would have saved with this critical scientific rationalization.

Forensics

Paré created and wrote “Reports in Court,” a legal writing procedure for courts [19]. He gave a methodical account of the violent death of internal organs. Because of this monumental work, Paré is also considered the father of modern forensics.

Prostheses

Paré dedicated a huge amount of time and effort to prosthetic design because he deemed them to be logical functional alternates. He dwelled in both limb and ocular prostheses [20]. Paré invented artificial eyes from enameled gold, silver, and glass, as well as from porcelain [12,21].

Bezoar stones

In the 16th century, bezoar stones were commonly believed to be able to cure the effects of any poison - a thought that was not backed by any evidence and hence challenged by Paré. He proved that this was indeed a myth when a thief who was condemned to be hanged, requested poison instead with the condition that he be given the bezoar stones after poison administration. Apparently, the thief’s request was accepted. Later, the thief died in agony as the bezoar stones, true to their nature, did not remove any effects of the poison [22].

“I treat them all like kings”

Paré treated all his patients equally and his treatment plan was always unbiased. Paré’s vision of patient-centric treatment was so unbiased that once when Charles IX requested that his treatment and cure be better than that afforded to the poor, Paré replied, “It’s impossible my lord because I treat them all just like kings” [13,17]. This shows the humble, impartial, and fair-minded nature of Paré as a surgeon.

Major milestones

The major milestones in the life of Ambroise Paré are given below in Table 1.

**Table 1:** Historical timeline of important milestones in the life of Ambroise Paré, the Father of Surgery.

Year	Milestone	References
1510	Ambroise Paré was born in Bourg-Hersent village in France	[1,3]
1525	Started apprenticeship at a surgeon-barber in Angers	[23]
1529	Started courses at the famous Barber-Surgery School at Hotel-Dieu Hospital, Paris	[4,12]
1533	Became a surgical resident at Barber-Surgery School at Hotel-Dieu Hospital, Paris	[10]
1536	Received the title of Master Barber-Surgeon. Also enrolled in the French Army and served under King Francis I (1536-38)	[4]
1537	Performed first elbow disarticulation (instead of cauterization)	[1,23]
1541	Qualified as Master Barber-Surgeon	[23]
1542	Recovered a tightly lodged bullet from the shoulder of Marshal of Brissac. Paré removed the bullet in seconds by reasoning that the patient should be placed in the same position as when the projectile hit him	[24]
1544	Started serving King Francois II (1544-1560)	[10]
1545	Published his first small book recommending the use of bland, soothing ointments for gunshot wounds and amputations	[25]
1545	Proved his surgical dexterity skills by retrieving a javelin trip from the Duke of Guise’s right zygoma	[24]
1547	Paré returned to Paris	[24]
1552	Paré named as the First Royal Surgeon, serving King Henri II	[12,23]
1552	Paré attempted and succeeded in the ligation of major vessels instead of cauterization with boiling oil	[12]
1554	Admitted to the Royal College of Surgeons, an institution which he was invited to head in 1567	[23]
1561-62	Paré published “Treaty of Human Universal Anatomy”	[24]
1562	Promoted to the post of Premier Surgeon to King Charles IX	[25]
1564-66	Paré followed Charles IX across France	[24]
1564	Paré published “Dix Livres de La Chirurgie”	[5,25]
1567	Bezoar Stone Experiment	[22]
1572	Published surgical book “Cinq livres de Chirurgie” with the first description of fracture of the head of the femur	[25]
1574	Paré elevated to the position of Premier Surgeon, Conséiller and Valet-de-Chambre under Henri III	[24,25]
1575	Oeuvres, the first collection of Paré’s accounts published	[9]
1585	Paré published a book titled “Apology and Journeys”	[5]
1590	Paré died peacefully in his Paris residence due to natural causes	[4]
1839	Sculpture of Ambroise Paré in Laval, France by the great 19th-century sculptor David d’Angers	[26]

## Conclusions

Ambroise Paré, a man of the Renaissance, was a great surgeon, philosopher, and compassionate human being with many accolades attributed to him. As a surgeon, he amalgamated his empirical observation and scientific reasoning to arrive at practices that are evidence-based thus leading to medical advancement in surgical practice. Paré believed in understanding the basics of any issue and emphasized the importance and relevance of understanding anatomical body structure, insisting that surgery must and should be based on anatomy. Paré had an incredible reputation as an innovator and was known as a person to come successfully out of hardships faced during his life. He was also a prolific writer and educator, as evidenced by his trove of articles and books that covered all aspects of surgery. Paré dedicated his final years of life to the publication of his books thus ensuring the vast practical knowledge and insights he gained from his substantial experience of evidence-based medicine are passed onto the next generation of surgeons. Above all, Paré, “the charitable surgeon,” was a humanist who always strived for peace, and excelled in treating all his patients equally with care, compassion, and with the utmost professionalism in an unpretentious and humble manner.

## References

[1] Drucker CB (2008). Ambroise Paré and the birth of the gentle art of surgery. Yale J Biol Med.

[2] Friedman SG (2018). Ambroise Pare: barber vascular surgeon. J Vasc Surg.

[3] Vinchon M (2009). Ambroise Paré, surgery, and obstetrics. Childs Nerv Syst.

[4] Popa CC, Marinescu AA, Mohan AG, Săceleanu MV, Ciurea AV (2018). Remember: Ambroise Paré (1510-1590) - message for young surgeons. Rom J Morphol Embryol.

[5] Hernigou P (2013). Ambroise Paré II: Paré's contributions to amputation and ligature. Int Orthop.

[6] Brockliss L, Jones C (1997). The Medical World of Early Modern France. https://global.oup.com/academic/product/the-medical-world-of-early-modern-france-9780198227502?cc=us&lang=en&.

[7] Paget S (1897). Ambroise Paré and His Times, 1510-1590. https://www.nejm.org/doi/abs/10.1056/NEJM189811241392111.

[8] Singer DW (1924). Selections From the Works of Ambroise Paré: With Short Biography and Explanatory and Bibliographical Notes. https://www.google.co.in/books/edition/Selections_from_the_Works_of_Ambroise_Pa/UVcuAAAAIAAJ?hl=en.

[9] Donaldson IM (2015). Ambroise Paré's accounts of new methods for treating gunshot wounds and burns. J R Soc Med.

[10] Splavski B, Rotim K, Boop FA, Gienapp AJ, Arnautović KI (2020). Ambroise Paré: his contribution to the future advancement of neurosurgery and the hardships of his times affecting his life and brilliant career. World Neurosurg.

[11] Park MT, Mignucci-Jiménez G, Houlihan LM, Preul MC (2022). Management of injuries on the 16th-century battlefield: Ambroise Paré's contributions to neurosurgery and functional recovery. Neurosurg Focus.

[12] Axioti AM, Geramani E (2018). Ambroise Paré - founder of modern surgery and pioneer of military medicine. J Surg Sci.

[13] Hernigou P (2013). Ambroise Paré's life (1510-1590): part I. Int Orthop.

[14] Banerjee AD, Nanda A (2011). Ambroise Paré and 16th century neurosurgery. Br J Neurosurg.

[15] Hernigou P, Hernigou J, Scarlat M (2021). Medieval surgery (eleventh-thirteenth century): barber surgeons and warfare surgeons in France. Int Orthop.

[16] Hernigou P (2013). Ambroise Paré III: Paré's contributions to surgical instruments and surgical instruments at the time of Ambroise Paré. Int Orthop.

[17] Hernigou P (2013). Other aspects of Ambroise Paré's life. Int Orthop.

[18] (1900). Neonatology on the Web. Anomalies and curiosities of medicine - prolificity. https://neonatology.net/classics/gould/gould.4.html.

[19] (2011). American College of Forensic Examiners. Forensic history timeline. https://web.archive.org/web/20110208142855/http://historyofforensics.com/.

[20] Hernigou P (2013). Ambroise Paré IV: the early history of artificial limbs (from robotic to prostheses). Int Orthop.

[21] Snyder C (1963). Ambroise PARE and ocular prosthesis. Arch Ophthalmol.

[22] Thompson CJS (1923). Poison Mysteries in History, Romance and Crime. https://archive.org/details/poisonmysteriesi00thomuoft#:~:text=Poison%20mysteries%20in%20history,%20romance%20and%20crime%20:%20Thompson,.

[23] Dunn PM (1994). Ambroise Paré (1510-1590): surgeon and obstetrician of the Renaissance. Arch Dis Child Fetal Neonatal Ed.

[24] Mauffrey C (2006). ‘Finally, I had run out of oil...’ Ambroise Paré and the birth of modern trauma surgery. Trauma.

[25] Shah M (1992). Premier Chirurgien du Roi: the life of Ambroise Paré (1510-1590). J R Soc Med.

[26] (2023). Linda Hall Library. Ambroise Paré. https://www.lindahall.org/about/news/scientist-of-the-day/ambroise-pare/.

